# The intestinal MUC2 mucin C-terminus is stabilized by an extra disulfide bond in comparison to von Willebrand factor and other gel-forming mucins

**DOI:** 10.1038/s41467-023-37666-8

**Published:** 2023-04-08

**Authors:** Pablo Gallego, Maria-Jose Garcia-Bonete, Sergio Trillo-Muyo, Christian V. Recktenwald, Malin E. V. Johansson, Gunnar C. Hansson

**Affiliations:** Department of Medical Biochemistry and Cell Biology, University of Gothenburg, SE-405 30 Gothenburg, Sweden

**Keywords:** Structural biology, Gastroenterology

## Abstract

The MUC2 mucin polymer is the main building unit of the intestinal mucus layers separating intestinal microbiota from the host epithelium. The MUC2 mucin is a large glycoprotein with a C-terminal domain similar to the MUC5AC and MUC5B mucins and the von Willebrand factor (VWF). A structural model of the C-terminal part of MUC2, MUC2-C, was generated by combining Cryo-electron microscopy, AlphaFold prediction, information of its glycosylation, and small angle X-ray scattering information. The globular VWD4 assembly in the N-terminal of MUC2-C is followed by 3.5 linear VWC domains that form an extended flexible structure before the C-terminal cystine-knot. All gel-forming mucins and VWF form tail-tail disulfide-bonded dimers in their C-terminal cystine-knot domain, but interestingly the MUC2 mucin has an extra stabilizing disulfide bond on the N-terminal side of the VWD4 domain, likely essential for a stable intestinal mucus barrier.

## Abstract (web summary)

The MUC2 mucin is a large, highly glycosylated polymer that builds the intestinal mucus. Here, the authors generate a high-resolution structural model of the 800 amino acid C-terminal dimer including its glycans. Stabilization is achieved by interdimer disulfide-bonds in both ends, essential for a stable mucus barrier.

## Introduction

The intestinal tract is efficiently organized to keep the epithelial surface free from bacteria as mediated by secreted mucus. In the colon, the inner mucus layer is impenetrable to bacteria whereas the continuous secretion of mucus and antibacterial molecules in the small intestine controls the bacterial load^1,2^. The formidable task of building the mucus barrier is dependent on the MUC2 mucin, the key building block of the mucus skeleton through its polymeric nature and high level of glycosylation. How this is accomplished is only partly known today and a better understanding will require detailed knowledge of the MUC2 molecular structure. As MUC2 and the other gel-forming mucins are highly glycosylated, there has been limited progress by traditional X-ray crystallography. Closely related to these mucins is the von Willebrand factor (VWF)^3^ which has provided some detailed structural insight of its C-terminal cystine-knot (CK) and part of the third von Willebrand assembly, the VWD’ and VWD3 domains^4,5^. The introduction of Cryo-electron microscopy (Cryo-EM) to study the molecular structure of large proteins has opened new possibilities as the structures of heavily glycosylated molecules can be analyzed. Recently, this approach elegantly revealed the complete structure of the N-terminal part of MUC2 in its packed intracellular form at low pH^6^.

**Fig. 1:**
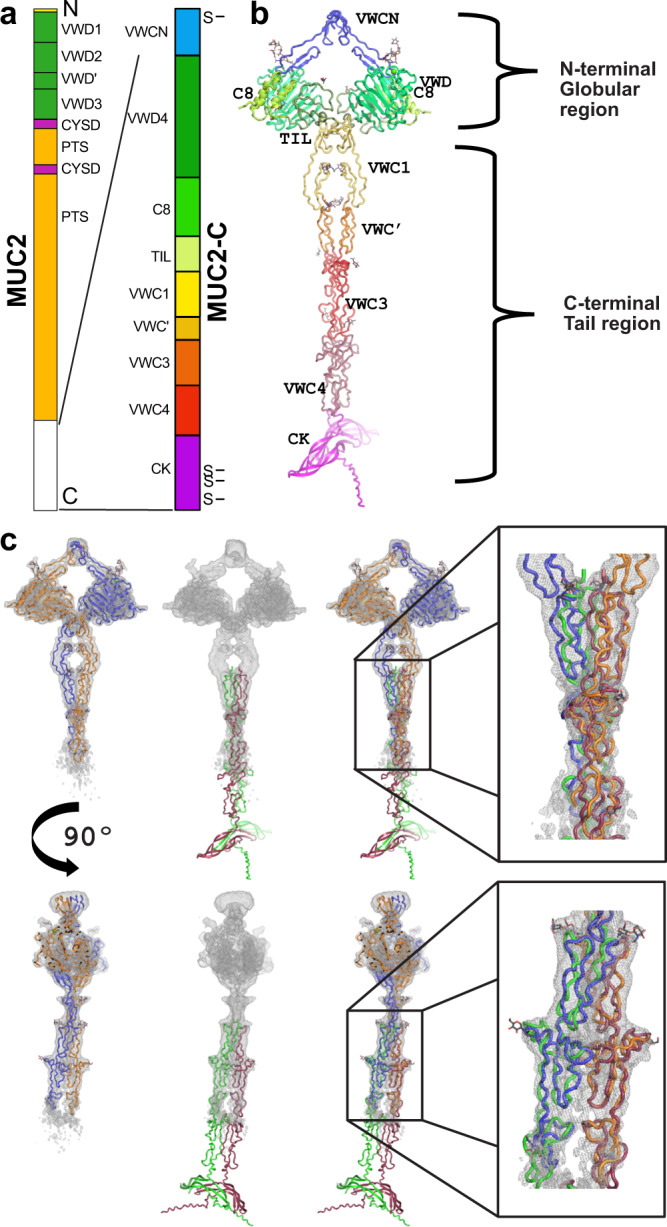
MUC2-C structural model. **a** The domain organization of the complete human MUC2 mucin and the expressed MUC2-C. **b** Colored cartoon representation of the full structure of MUC2-C assembled as a dimer. The different domains are colored as in **a**. **c** Frontal and lateral views of the combined Cryo-EM and AlphaFold2 models of MUC2-C. The r.m.s.d. of the superposition of the Alphafold2 model and the Cryo-EM model is 3.5 and 3.7 for each monomer. The r.m.s.d. deviation was calculated considering the Cα from the VWC’ to the VWC3. In orange and blue, the two different monomers of the MUC2 C-terminal model obtained by Cryo-EM. The Cryo-EM map is represented as a grey net. In green and red the two different monomers of MUC2 C-terminal model obtained by AlphaFold2.

The MUC2 mucin consists of more than 5000 amino acids, with a molecular mass of more than 2 MDa when the extensive *O*-glycosylation is included. The MUC2 mucin were originally partly sequenced by Gum et al.^7^, but the two central highly repetitive sequences primarily made up by the amino acids threonine, serine, and proline (PTS) were only recently sequenced^8^. The C-terminal part of MUC2 is made up by a VWD assembly, a series of VWC-domains and a C-terminal CK (Fig. 1a). The structure of the CK domain is known from other molecules containing this domain where it forms a dimer by three disulfide-bonds as in VWF^4^. A low-resolution image of the MUC2-C closely related MUC5B C-terminal has been published^9^, but currently no high-resolution information is available for the mucins or VWF. Here, we present a model of the C-terminal part of MUC2 (MUC2-C) revealed by combining Cryo-EM and AlphaFold modeling which show an elongated dimer held together by covalent disulfide bonds in both ends.

## Results

### Purification of MUC2-C protein and analysis of its disulfide bonds

The human MUC2 mucin has a variable central PTS sequence of which one variant has been completely sequenced^8^. This sequence was used as the basis for the amino acid numbering and expression of the 774 amino acid MUC2-C in CHO cells (Fig. 1a). Gel filtration of the purified protein indicates MUC2-C migration comparable to a globular protein with a molecule mass over 600 kDa (Supplementary Fig. S1). Polyacrylamide gel electrophoresis shows a band of around 300 kDa under non-reduced conditions and after reduction a band of about 150 kDa, as expected demonstrating that it was a covalent dimer. MUC2-C contains 80 cysteine amino acids and these were analyzed for the presence of free non-disulfide-bonded cysteines. This was studied by a differential alkylation approach by first reacting it with N-ethylmaleimide (NEM) followed by reduction with dithiothreitol (DTT), iodoacetamide (IAA)-labeling, proteolysis, and mass spectrometric analysis. Most cysteine-containing peptides were alkylated with IAA and not with NEM showing derivatization after reduction. These results indicate that most cysteine residues were oxidized and thus involved in the formation of disulfide bridges. For a few peptides, different spectra were obtained where the cysteines were either labeled by either IAA or NEM (Supplementary Fig. S2). However, the intensity of peptides showing the NEM-label was two orders of magnitude lower than the corresponding IAA-labeled peptides, suggesting sample preparation artefacts or instability of some disulfide bonds. We concluded from these results that the cysteines were oxidized and involved in the formation of disulfide bonds, a conclusion supported by Cryo-EM results as discussed below.

### The MUC2-C structure reveals disulfide-bonded dimers in both ends

MUC2-C was analyzed by Cryo-EM (Supplementary Table S1). As described previously for MUC5B and VWF using negative staining, the C-terminal tail of these related molecules show high flexibility limiting the possibility to generate complete 3D maps^9–11^. The domain names follow that used for the VWF (Fig. 1a)^12^. Using Cryo-EM, it was possible to obtain structural information of MUC2-C from the VWCN domain to the VWC3 domain at an average resolution of 3.4 Å (Supplementary Table S1 and Fig. S3). Information regarding the local resolution of VWCN is shown in Supplementary Fig. S4. To complete the structure of MUC2-C, we used AlphaFold2 to predict a model from the VWC3 to the C-terminal end including the CK domain similar to the published structure of VWF-CK^13^. The MUC2-C dimer model shows two regions: an N-terminus with a compact globular peptide chain and a C-terminal tail with the domains in tandem organized into a stalk that ends with the CK domain (Fig. 1b). Figure 1c shows how the Cryo-EM and AlphaFold2 models were combined to form MUC2-C.

The MUC2-C homodimer globular region in the N-terminal end presents a C2 symmetry, whereas the C-terminal tail had a non-regular double and twisted stalk conformation. The overall structure resembled a ‘T’, similarly to the low-resolution model of the MUC5B C-terminal dimer^9^. The individual domains were clearly separated and the organization of the stalk sheds light on the behavior of the VWC domains. The MUC2-C dimeric structure was stabilized by three disulfide bonds in the CK dimerization interface as in VWF^14,15^. Interestingly, MUC2-C contained one additional interdimer disulfide bond in its N-terminus. The VWF sequence was aligned with the MUC2 and MUC5B mucins and the previously suggested disulfide pairs for VWF^11^ combined with the presently identified ones gave a slightly modified map of the disulfide bond organization in the C-termini of these proteins (Supplementary Fig. S5).

### N-terminal globular region of MUC2-C

**Fig. 2:**
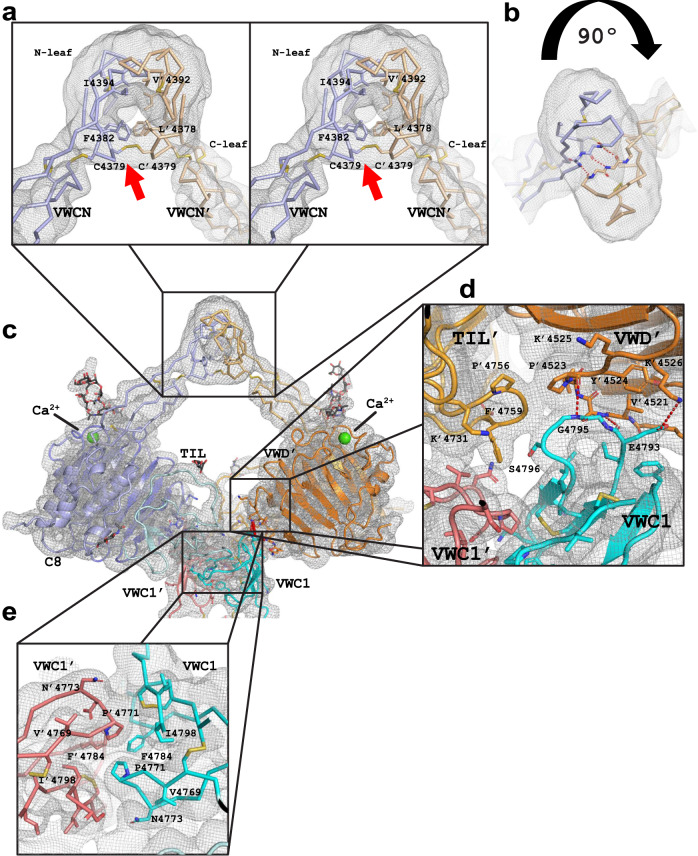
The N-terminal globular region of the MUC2-C dimer. **a** Frontal representation in stereo of the homodimeric interface of the domain VWCN. The monomers of the dimer are colored in blue and orange. Cryo-EM map is represented as a grey net. The red arrow points to the C4379-C4379’ disulfide bond. **b** Top view of the homodimeric interface of the domain VWCN. **c** The globular region of the MUC2-C. In orange, one monomer with the domains VWCN, VWD, C8, and TIL followed by the N-leaf of the VWC1 of the same monomer, in red. In blue, the other monomer representing the same domains followed by the corresponding N-leaf of the VWC1 in cyan. **d** Interface between the VWD (orange), TIL (bright orange) and VWC1 (red) of one monomer interacting with the VWC1’ (cyan) of the other monomer. The side chains of the amino acids playing a major role are represented and labeled. **e** The dimeric interface governed by the leaf region of the N-Leaf of the VWC1 (red) of one monomer versus the corresponding N-Leaf of the VWC1’ (cyan) of the other monomer. The amino acids driving the interaction are represented and labeled.

The N-terminal globular region starts with the domain previously named VWD4N in VWF^12^. The MUC-2C structure presented here shows that this domain has a VWC domain fold, which we renamed VWCN. This domain is followed by a typical VWD assembly similar to the VWD3 assembly of VWF^5^ and MUC2^16^ (Fig. 1a), containing a VWD domain, a C8 domain, and a TIL domain (Fig. 1b). As described for MUC5B^9^, the dimerization of these regions shows a C2 symmetry and the dimeric interactions are formed by two main interfaces. The first confined to the VWCN domain and the second to the VWC1 domain (Fig. 2).

The VWCN domain dimer interface consists of the hydrophobic amino acids I4394, V4392, F4382, L4378 of each chain (Fig. 2a, c) and an antiparallel β-sheet interaction between the β-strands of from T4391 to E4393 (Fig. 2b). The dimer is stabilized via a unique intermolecular disulfide bond between the C4379 of both monomers (Fig. 2a, red arrow) to form the closed N-terminal arrangement of the MUC2-C. Interestingly, this interaction is not covalently stabilized in the other gel-forming mucins or the VWF as all lack this cysteine^17^. The second interaction involves the first VWC domain (VWC1) and occurs via two interfaces. First, the amino acids G4795 and E4793 of a loop in the VWC1 of each monomer create hydrogen bond interactions with the corresponding VWD domain of the other monomer (Fig. 2d). The second dimeric interface is formed via the interaction of VWC1 with the corresponding VWC1 of the other monomer (Fig. 2e). This hydrophobic interaction is mediated by the P4771, F4784, and I4798 amino acids of the VWC1 N-terminal.

The VWD4 contains the GDPH motif at amino acids 4436–4439, which can be cleaved autocatalytically. In MUC2-C, this is not cleaved during biosynthesis in CHO cells and is only cleaved after exposure to acidic conditions^18^. The Asp-Pro peptide bond is thus intact in our model.

### VWC domains

**Fig. 3:**
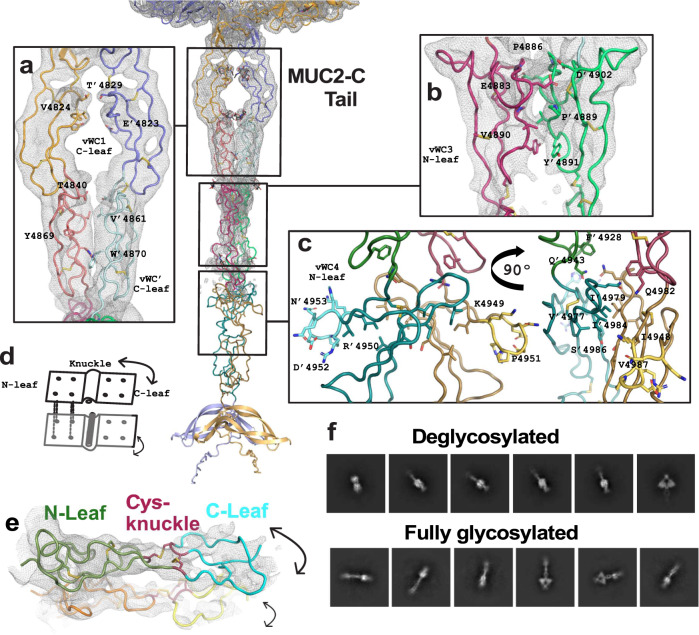
The tail region of the MUC2-C dimer. The Cryo-EM maps represented as a grey net. **a** The dimeric structure formed by the C-Leaf of VWC1 (blue and orange) and VWC’ (salmon and light cyan) of MUC2-C with the nearby amino acid side chains represented. **b** The dimeric interface formed by the N-Leaf of VWC3 (magenta and green) with the key amino acid side chains for the interface represented. **c** The dimeric interface formed by the N-leaf of vWC4 (cyan and brown). In the left panel, frontal view of the RPD sequence at the position for the RGD motifs exposed at both sides (cyan and yellow). In the right panel, lateral view of the dimeric interface with key amino acid side chains for the interface represented. **d** Schematic representation of the mechanics and structural functionality of a single VWC dimer. The N-leaf interactions hold the dimer, while the C-leaf has free movement and both leaves are separated by a knuckle. **e** The dimer of VWC3 of MUC2-C. Represented in green is the N-leaf and in cyan the C-leaf, the second monomer orange and yellow, respectively. The two disulfide-bond pairs acting as a knuckle are represented in magenta. A grey net represented the Cryo-EM map. **f** Representative 2D classifications of MUC2-C fully *N*-glycosylated and with a single GlcNAc at each *N*-glycan site (called deglycosylated).

The VWC domain consists of two regions, one at the N-terminus (N-leaf) consisting of a β-hairpin  followed by three β-strands organized in an antiparallel β-sheet and a C-terminal part (C-leaf) with three β-strands arranged similarly (Fig. 3). Both subdomains are linked to each other via the peptide chain and two disulfide bonds that act as a bendable knuckle, Cys-knuckle (Fig. 3d, e), previously observed by NMR for the VWD4 of VWF^19^. This knuckle allows the VWC to bend and is important for internal domain flexibility. Interestingly, for all domains in MUC2-C the N-leaf subdomain mediates domain dimerization (Figs. 2a, e and 3b, c). In contrast, the C-leaf subdomain does not dimerize at all, with only minor interactions found in VWC1 and VWC’ (Fig. 3a).

### C-terminal tail of MUC2-C

The C-terminal tail region (Fig. 1a) begins with the second subdomain of the VWC1 domain (C-Leaf). The VWC1 is followed by VWC’, a truncated VWC domain, VWC3 and VWC4 domains and the C-terminal end with a typical CK domain (Figs. 1 and 3). The gel-forming mucins have an identical domain distribution except for MUC6, which lacks VWD4 and the VWC domains. In contrast, the VWF protein has an additional VWC2 domain before VWC’ and two additional domains (VWC5 and VWC6) after VWC4 (Supplementary Fig. S5).

In the literature, there has been ambiguity in the description of the domains of the C-terminal region of mucins and the VWF due to the complexity of the sequence and the distribution of disulfide bonds. Our structural information shows that there is no VWB domain, but rather a serial repetition of VWC domains, disturbed by the truncated VWC’ domain only containing the C-leaf part and the two disulfide bonds acting as a knot (Fig. 3d, e).

The C-terminus of MUC2-C shows high flexibility due to several bending points. These are located between each VWC domain and within the Cys-knuckle subdomain. During particle selection and 3D volume reconstruction, this flexibility made it impossible to extend the map beyond VWC3 of the fully glycosylated MUC2-C (Figs. 1c and 3e, f). The Cryo-EM map loses intensity after the Cys-knuckle of VWC3. To obtain further understanding, we expressed the MUC2-C in the presence of kifunensine to obtain *N*-glycans of the high-mannose type. Treatment with EndoH resulted in MUC2-C with only a single GlcNAc at the *N*-glycan sites. When this less glycosylated MUC2-C was analyzed by Cryo-EM (Fig. 3f, deglycosylated), it was only possible to resolve the VWC1 domain suggesting that the *N*-glycans restrain the flexibility of the MUC2-C tail. Alternatively, the less defined tail of the deglycosylated MUC2-C could be caused by its lower mass and increased difficulties in image analysis.

The VWC4 domain of VWF mediates the interaction between VWF and integrin α_IIb_β_3_ via an RGD sequence motif ^20,21^. This interaction occurs through a β-hairpin located in the N-leaf of the VWC4 domain. The VWC4 of MUC2-C does not contain this RGD sequence, but the glycine has been replaced by a proline (4950-RPD-4952) which is exposed to the surface (Fig. 3c).

The MUC2-C terminates in the characteristic CK domain with a strong dimerization interface with three disulfide bonds^4,22^. The CK domain is followed by an unstructured tail including two double arginine motifs, previously shown to be cleaved off by furin^23^.

### Glycosylation of MUC2-C

**Fig. 4:**
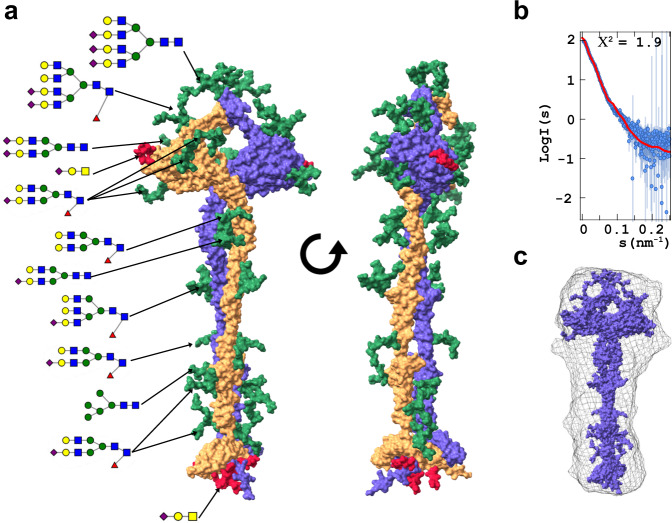
Complete model of the MUC2-C including glycans and small angle X-ray scattering analysis. **a** The MUC2-C structural model (Fig. 1) were complemented by the most abundant *N*-glycans (green) and *O*-glycans (red) at each site^24^. **b** Comparison between calculated scattering curve from the complete MUC2-C as in **a** (red line) and the experimental SAXS (blue circles). **c** SAXS ab initio averaged model of compared as a grey cloud with the final model (a) inserted.

The generated Cryo-EM/AlphaFold MUC2-C model (Fig. 1) contained unstructured loops. Using the Modloop program, these were modeled to complete the structure of the polypeptide chain. We have previously made a detailed mass spectrometric analysis of the MUC2-C glycans and their localization in CHO-K1-produced MUC2-C^24^. Using this information, we added the most abundant glycans with the CHARMM-GUI program at each attachment site of the MUC2-C model. All information was finally combined into a more complete model of MUC2-C (Fig. 4a) (model found at [www.medkem.gu.se/mucinbiology/structures/]). The *N*-glycans were of di- to tetra-antennary type with a tendency for having the larger *N*-glycans found on the VWCN and VWD4 domains and the less complex ones on the MUC2-C tail. A few *O*-glycans were observed in the mass spectrometry analyses, especially in the CK-domain^24^. The tail made up of VWC domains has equally spaced *N*-glycans whose presence suggest that they may restrict its flexibility by the proximity of the glycan chains that can easily result in steric collisions.

SAXS analyses (Fig. 4b and Supplementary Fig. S6 and Table S2) of the recombinant MUC2-C confirmed a homodimer in solution with a radius of gyration (Rg) of 8.35 nm and an estimated molecular mass of around 208 kDa in comparison to the dimer theoretical mass of 240 kDa including the most abundant glycans. The Kratky plot (Supplementary Fig. S6d) does not show a classical globular protein profile, but rather a displaced maximum suggesting a mixture of globular and rod structures with flexible or partially disordered regions. Furthermore, the pairwise distance distribution (p(r)) function (Supplementary Fig. S6c), showed a bimodal function indicating a multidomain molecule with a maximum particle dimension (Dmax) of 32 nm. The calculated scattering curve of MUC2-C (Supplementary Fig. S6a) agrees closely with the experimental results (Fig. 4b). Finally, the SAXS ab initio averaged model was shown to accommodate the complete glycosylated dimer structure of MUC2-C (Fig. 4c).

## Discussion

The C-terminal parts of the mucin-VWF family of proteins have previously only been described at low resolution for WVF and MUC5B^9,10^, except for the VWF CK domain whose crystal structure has been resolved^4^. This study describes the Cryo-EM generated N-terminal structure of the MUC2-C including the detailed structure of the VWD4 assembly and the first VWC domains. The molecular structures described here should be similar to that of the VWF, MUC5AC, and MUC5B. The MUC2-C structural model will therefore provide key structural insights into these other molecules. Most of the C-terminal VWC domains were impossible to observe in Cryo-EM due to their flexibility. A full MUC2-C structural model was assembled using the Cryo-EM results combined with AlphaFold2 multimer *in-silico* prediction based on the previous VWF-CK model^4^. Using our previous detailed analyses of the MUC2-C glycans^24^, it was possible to build a complete model of the MUC2-C, which was compatible with the SAXS scattering profile (Fig. 4a). This model shows that the MUC2-C consists of an elongated dimeric stalk structure coated with spaced *N*-glycans.

The detailed structure of MUC2-C reveals that the region between the VWD assembly and CK consists entirely of VWC domains where one is truncated. In contrast to previous suggestions, the MUC2-C does not contain any VWB domains. Sequence alignments (Supplementary Fig. S5) suggest a similar arrangement among the gel-forming mucins, except MUC6. The VWF is organized as VWC1-VWC2-VWC′-VWC3-VWC4-VWC5-VWC6 while MUC2-C and MUC5B-C lack the VWC2, VWC5, and VWC6 domains (Supplementary Fig. S5).

The VWD assembly including the VWD domain is not only found in the closely related gel-forming mucins and VWF, but also within a larger group of proteins such as FCGBP, Otogelin, Tectorin, and sco-Spondin^3^. The VWD assembly appeared early in evolution^3^.

The VWF-C forms a dimeric bouquet at the low pH of the secretory pathway which is pulled out by the mechanical forces generated by the blood flow at physiological pH^10^. The MUC5B-C forms a similar dimeric bouquet, which seems to be maintained irrespectively of pH^9^, although it is likely stretched open if subjected to pulling forces. This is in contrast with the MUC2-C which possesses an extra Cys (4379) located in the VWCN domain at the N-terminal side of VWD4, and is mediating an extra intermolecular disulfide-bond between the two MUC2-C chains. This indicates that, unlike the other gel-forming mucins, the MUC2-C is covalently linked at both ends, via intermolecular disulfide bridges involving both the VWCN and CK domains. Thus, the MUC2 mucin cannot be pulled out and extended, as is the case for MUC5B and VWF. This extra disulfide bond is probably vital for maintaining the mucin covalent polymeric network in the protease-rich milieu of the intestine. We have previously suggested that cleavages in the MUC2 C-termini were responsible for mucus expansion, without disassembly of the covalent mucin network, during the transition from the colon inner to outer mucus layer^25^. However, the discovery of this extra disulfide-bond in the N-terminal end of MUC2-C makes this hypothesis unlikely. This also means that the two central highly *O*-glycosylated PTS domains of the complete MUC2 are covalently cross-linked by disulfide-bonds in both ends, at the *N*-terminal end within the VWD3 domain^6,26^ and in the VWCN domain as shown here. This may suggest that the large portions of the complete MUC2 N- and C-termini before and after the intermolecular disulfide-bonds are not required for holding the MUC2 mucin polymeric network together. However, these parts of MUC2 are critical for the intracellular assembly and packing of the mucin and are likely crucial for interactions between mucins and other molecules in the mature mucus gel.

Interestingly, the N-terminal globular region of MUC2-C is decorated with larger *N*-glycans, up to tetra-antennary types, than the simpler *N*-glycans in the MUC2-C tail region. The presence of these glycans together with the tightly folded disulfide-bond stabilized MUC2-C globular region can be suggested to protect this part against proteolytic cleavages by both endogenous and microbial proteases, both abundant in the harsh conditions of the colon. This prediction is supported by recent mass spectrometric analyses of MUC2 in colon mucus from *Tgm3*^*−/−*^ mice where MUC2-C was, together with the CysD2 domain, better preserved than the N-terminal parts of MUC2^27^.

If almost 800 amino acids of the MUC2 C-terminal end are not required for maintaining mucin covalent polymers, what other functions remain to be discovered? Building a functional mucus will require numerous interactions between mucins and other proteins contributing to the properties of the mucus gel. Suggestions can be gained from protein interactions of the VWF, where the platelet α_IIb_β3 integrin binds the RGD loop at VWF amino acid 2510. This loop is shortened and altered to RPD in MUC2-C, but still exposed. There is a promiscuity of the RGD binding motifs^28^, but replacing glycine with proline should have dramatic effects and may suggest other interaction partners than integrins.

The long and extended nature of the MUC2-C tail may however also suggest its involvement in other unexplained interactions. The inner mucus layer of the colon remains attached to the epithelial surface until it is transformed into the outer mucus layer^1^. In the small intestine a protease, Meprin β, is required to detach the mucus from the epithelial surface, although the only cleavage site identified was in the MUC2 N-terminus^29^. Both the MUC5B mucin and VWF are unfolded while being pulled out from the secreting cell by a liquid flow at the cell surface^12,30^. This process requires that the molecules remain anchored in the cell. Potential interactions with the C-terminal tail of these molecules require further molecular exploration.

The detailed molecular information of the C-terminal part of the MUC2 mucin including its glycans reveals key structural characteristics and unique features of MUC2 that will help explaining the properties of intestinal mucus. These observations are applicable also to the other mucins and VWF. This knowledge should open for understanding more of the function of these proteins and new interaction partners, likely of high medical importance.

## Methods

### Cloning, expression vectors and DNA preparations

The pcDNA3.1(+) plasmid was used as a template for inserting the MUC2-C (NCBI reference: MH593786.1^8^ (protein AZL49145.1) containing the amino acids 4356–5130 with an N-terminal IGk signal sequence, a 6 His-tag and the 3C precision cleavage tag in that order from the N-terminal. The mammalian expression vector was transformed through electroporation using XL1 Blue Electrocompetent cells (Agilent). The transformed cells were selected with the corresponding antibiotic and grown overnight in LB Broth Base (Invitrogen). DNA was prepared using PureLink™ HiPure Expi Plasmid Megaprep Kit (Thermo Fisher Scientific).

### Expression and purification of recombinant MUC2-C

Using the NovaCHOice transfection kit (Merck Millipore), the recombinant MUC2-C expression vector was transiently transfected into CHO-S cells growing in 800 ml flasks and FreeStyle CHO growth medium (Gibco) without serum. The extracellular recombinantly expressed protein was collected 72 h after the transfection by centrifuging the cell media at 500 × g and 6000 × g and discarding the cell pellet. To obtain the high-mannose recombinant MUC2-C the expression was done in presence of 1 μg/ml kifunensine (Toronto Research Chemicals).

The recombinant protein was purified from the supernatant by Immobilized metal affinity chromatography (IMAC) method using Ni-Sepharose® Excel (Cytiva) in a loaded gravity column (BioRad). The loaded resin was washed with 300 mM NaCl, 20 mM HEPES pH 7.4 and 40 mM imidazole, and the protein was eluted with 300 mM NaCl, 20 mM HEPES pH 7.4 and 300 mM imidazole. For the high-mannose MUC2-C the elution was dialyzed over-night against 150 mM NaCl, 20 mM MES, pH 5.5, and treated with EndoH (Roche) glycosidase at 4 °C. The products were then purified by Size Exclusion Chromatography (SEC) using an Äkta HPLC system (GE Healthcare) with a HiLoad 16/600 Superdex 200 column (GE Healthcare) in 150 mM NaCl, 20 mM HEPES, pH 7.4.

### Analysis for free disulfide bonds

The pure MUC2-C protein was incubated with a 1000-fold molar excess of NEM for 2 h in PBS, pH 7.2. Free NEM was removed by five washing steps through a 10 kDa ultrafiltration unit and the buffer was exchanged to TBS, pH 8.0. Freshly prepared DTT, 10 mM, was added and disulfides were reduced for 30 min at 60 °C. After cooling the reaction mixture to RT, 25 mM iodoacetamide (IAA) was added and the solution incubated for 30 min RT. The protein was digested with trypsin and differentially labeled cysteine residues identified by mass spectrometry. Buffer and salt contaminants were removed by in-house built C-18 stage tips and the peptide mixture subjected to LC-MS/MS analyses using a Q-Exactive HF instrument (Thermo). Analysis was performed with the MASCOT and PEAKS search engines using the following parameters: (i) parent ion mass tolerance, 5 ppm; (ii) mass tolerance of fragment ions, 0.2 Da; (iii) cleavage C-terminal of K and R unless followed by P; (iv) maximum two missed cleavages; (v) variable modifications for Cys residues were carbamidomethylation with IAA as well as NEM derivatization; (vi) oxidation of methionine as variable modification.

### Cryo-EM grid preparation and data acquisition

The MUC2-C recombinant protein, fully glycosylated and partly deglycosylated, were incubated overnight, at 1 mg/ml in a buffer solution containing 150 mM NaCl, 20 mM HEPES, and 5 mM CaCl_2_ at pH 7,4 at 4 °C. A 2 µl aliquot of the MUC2-C glycosylated protein solution was loaded onto a Quantifoil R1.2/1.3, 300 mesh gold grid that was previously glow discharged with a negative charge at 15 mA for 40 s. The grid was plunge frozen in liquid ethane cooled by liquid nitrogen using a Vitrobot plunger (Thermo Fisher Scientific) at 100% humidity. For the partly glycosylated MUC2-C, an identical protocol was used before loading the sample onto a Quantifoil R1.2/1.3, 300 mesh copper grid.

Cryo-EM data were collected on a Titan Krios G2 transmission electron microscope (Thermo Fisher Scientific) operated at 300 kV. Parameters for Cryo-EM are described in Supplementary Table S1. Movies of 80 frames each were recorded on a Gatan K3 detector (Gatan). Movies were recorded in counting mode at a nominal magnification of ×105,000 zoom, corresponding to a physical pixel size of 0.86 Å. The total dose rate was set to 80.15 e^−^/Å^2^. Nominal defocus range was −1 to −2 µm. EPU software (Thermo Fisher Scientific) in Aberration-free image shift (AFIS) mode was used for automated data collection, in which two images were collected from each hole.

### Cryo-EM data analysis

Cryo-EM movie data processing and image fitting were done using CryoSPARC software v3.2^31^. Patch motion correction and patch CTF estimation were done over a total of 13,210 and 6,406 movies collected for the MUC2-C glycosylated and partly deglycosylated proteins, respectively. An initial particle picking was done using the Blob Picker followed by particle extraction, 2D classification and 3D heterogeneous refinement. The selected particles were used to create an AI model for novel particles picking with topaz^32,33^. Two final sets of particles were obtained for each conformation: 369,150 particles for the glycosylated and 387,288 for the partly deglycosylated MUC2-C. Both sets of particles and the corresponding 3D volumes were refined using the 3D and CTF refining tools, ending with a final local refinement job. As the volume for the contracted confirmation presented a C2 symmetry, this parameter was set for the refinement of this conformation.

An initial backbone structure for the MUC2 C-terminal glycosylated and partially deglycosylated protein was created by manual fitting with UCSF Chimera^34,35^ a monomer of the MUC2 model D3 dimer (PDB ID 6RBF)^16^ in each corresponding VWD4 module and additional fitting each VWC domain volume to the corresponding VWF VWC4 domain (PDB ID 6FWN)^19^. The initial models were finally manually built using Coot^36^, aided by the autobuild tool of Phenix^37^ and using the NCBI reference: MH593786.1 sequence^8^. The final structures refinement validation for each conformation was assessed by Molprobity^38^. The structure analysis and figures were generated using PyMol and UCSF Chimera. The structures are PDB deposited with ID 7QCL for the glycosylated MUC2-C and 7QCU for the partly deglycosylated MUC2-C.

### In-silico structure prediction using AlphaFold2 and a composite model

The prediction for the truncated and dimeric model for the construction of the MUC2-C from the vWC3 domain to the CK domain (amino acids 4874–5130) was obtained using the AlphaFold2 software and its multimeric models^13^. The algorithm was run through the Deep mind Google Collaboratory (Colab) notebook template application and servers. (https://colab.sandbox.google.com/github/deepmind/alphafold/blob/main/notebooks/AlphaFold.ipynb).

To build a complete model of the MUC2-C, the non-assigned loops from the Cryo-EM/AlphaFold model were computed using Modloop^39^ and the most abundant glycan were added at each glycosylated site according to previous analyses by mass spectrometry^24^ using CHARMM-GUI^40^. For the areas where electron densities corresponding to *N*-glycans were possible to observe, only the first attached GlcNAc was maintained according to the density map and extension was made according to CHARMM-GUI^40^. A PDB file with the complete MUC2-C is available at www.medkem.gu.se/mucinbiology/structures.

### Small angle X-ray scattering

Small Angle X-ray Scattering (SAXS) measurements were recorded at 25 °C with a momentum transfer range from 0.05–6.2 nm^−1^ and *λ* = 0.0826 nm, at BioSAXS beamline BM29 at the European Synchrotron Radiation Facility (ESRF), Grenoble, France. SEC-SAXS was collected during all the elution in 25 mM HEPES pH 7.4, 100 mM NaCl, and 10 mM CaCl_2_ according to the parameters described in Supplementary Table S2, including references to software packages used. Data reduction and 1D scattering intensities of the sample were done at the beamline BM29. Frames selection, averaging, buffer subtraction and extrapolation were performed using CHROMIXS and primusqt from the ATSAS 3.0.0 package. The radius of gyration (Rg), forward scattering (I(0)), maximum particle dimension (Dmax) and the distance distribution function were determined with GNOM (SI Appendix Table 2). The molecular mass was obtained using the Bayesian inference approach with the ATSAS package. Initial ab initio models were obtained by running dummy atom modeling DAMMIN. The models were aligned, compared, averaged and filtered using DAMAVER. The final model was used to manually fit the MUC2-C dimer as obtained by Cryo-EM, AlphaFold, and glycan additions using UCSF ChimeraX. The SAXS results are deposited to SASBDB with accession number SASDPL4.

### Reporting summary

Further information on research design is available in the Nature Portfolio Reporting Summary linked to this article.

## Supplementary information

Supplementary Information

Reporting Summary

## Data Availability

The protein sequences were MUC2 from Svensson et al. Scientific Reports 8, 17503(NCBI reference: MH593786.1), VWF (Uniprot P04275, NCBI reference NM_000552.4), and the MUC5B (UniProtKB/Swiss-Prot: Q9HC84.2). The cryo EM structures and maps have been deposited in The Worldwide Protein Data Bank archive [http://www.wwpdb.org/] under accession codes 7QCL and 7QCU. The previously published PDB structures referred to in the text are found under accession codes 6RBF and 6FWN. The cryo EM maps are deposited to the Electron Microscopy Data Bank (https://www.ebi.ac.uk/emdb/) under accession codes EMD-13896 and EMD-13899. SAXS data are deposited to the Small Angle Scattering Biological Data Bank [https://www.sasbdb.org/] under accession code SASDPL4. The complete model of MUC2-C including the glycosylations will be available at the SASBDB and at [www.medkem.gu.se/mucinbiology/structures]. The authors declare that all relevant data supporting the findings of this study are available within the paper, its supplementary information files, and at the stated open depositories.
