# Supplementary material for: The intestinal MUC2 mucin C-terminus is stabilized by an extra disulfide bond in comparison to von Willebrand factor and other gel-forming mucins
Source: Nat Commun. 2023 Apr 8;14:1969. doi: 10.1038/s41467-023-37666-8 (PMC10082768; doi:10.1038/s41467-023-37666-8)
Supplement: Supplementary file 1 — Supplementary Information [file 41467_2023_37666_MOESM1_ESM.pdf]

SUPPLEMENTAL MATERIAL

The intestinal MUC2 mucin C-terminal is stabilized by an extra disulfide bond in comparison to von Willebrand factor and other gel-forming mucins.

Pablo Gallego, Maria-Jose Garcia-Bonete, Sergio Trillo-Muyo, Christian Recktenwald, Malin E.V. Johansson, and Gunnar C. Hansson

Supplemental material:

Supplementary Table S1 Cryo-EM/PDB information Page 2
Supplementary Table S2 SAXS data collection Page 3
Supplementary Figure S1 Purification of MUC2-C Page 4
Supplementary Figure S2 Mass spectra analysis of Cys residues Page 5
Supplementary Figure S3 Structure and resolution determination Page 6
Supplementary Figure S4 Cryo-EM of the WVD Page 7
Supplementary Figure S5 Sequence alignments MUC2, MUC5B and VWF Page 8
Supplementary Figure S6 SAXS results Page 9
References Page 10

Supplementary Table S1. Cryo-electron microscopy parameters for the analysis of MUC2-C.

**Structure**	**MUC2-C Glycosylated**	**MUC2-C Deglycosylated**
**Data Accession**		
PDB	7QCL	7QCU
EMDB	EMD-13896	EMD-13899
**Data Collection**		
Microscope	TFS KRIOS	TFS KRIOS
Voltage (kV)	300	300
Detector	GATAN K3 Detector	GATAN K3 Detector
Pixel Size (Å)	0.86	0.86
Electron exposure ($e^-/Å^2$)	79	79
Defocus range (μm)	-1.0 to -2.0	-1.0 to -2.0
Micrographs collected	13,210	6,406
**Reconstruction**		
Software	CryoSPARC (v3.2)	CryoSPARC (v3.2)
Micrographs used	13,210	6,406
Particles used in refinement	369,150	387,288
Symmetry imposed	C1	C1
Overall resolution (Å) FSC=0.143 (masked)	3.36	3.25
Map sharpening B-factor ($Å^2$)	-94	-115
Local resolution range (Å)	2.2 ~ 7.0	2.2 ~ 5.0
**Model Refinement**		
Software	Phenix (v1.19.2-4158)	Phenix (v1.19.2-4158)
Non-hydrogen atoms	9,884	7,069
Protein residues	1092	888
Ligands	127	20
Average B factors ($Å^2$)		
Protein	39.98	40
Ligands	30.98	37.56
R.M.S. deviations		
Bond length (Å)	0.009	0.012
Bond angle (°)	1.781	2.237
Ramachandran statistics (%)		
Outliers	0.09	0
Allowed	9.94	6.88
Favored	89.96	93.12
MolProbity score	3.21	2.97
Model vs. Map FSC		
FSC=0.5 (masked, Å)	4.1	3.7

Supplementary Table S2. SAXS data collection, processing and modeling statistics of MUC2-C dimer.

**Data collection parameters**			
Beamline	ESRF BM29 , Grenoble, France		
Beam geometry (μm²)	200 x 200		
Wavelength (nm)	0.08266		
Detector	Pilatus 2M in vacuum		
Detector distance (m)	2.867		
q range (nm^{-1})	0.05 - 6.2		
Sample environment	Quartz glass capillary, 1mm diameter		
Exposure time (s) per frame	1		
Temperature (°C)	25		
Mode	SEC-SAXS		
**SEC-SAXS parameters**			
Column	Agilent Bio SEC-3 300Å		
Injected volume (μl)	100		
Concentration (mg/ml)	1.8		
Flow rate (ml/min)	0.3		
Solvent composition	25mM Hepes 100mM NaCl 10mM $CaCl_2$ pH 7.4		
Frames	Total	Buffer	Protein
	540	57	11
**Structural parameters**			
Guinier analysis			
I(0) (cm^{-1})	105.95 +/- 1.01		
Rg (nm)	8.35 +/- 0.18		
q Rg-range	0.05 – 0.15		
Total quality estimate (AutoRg)(1)	0.77		
P(r) analysis			
I(0) (cm^{-1})	108.08		
Rg (nm)	9.13		
q-range (nm^{-1})	0.05-1.28		
D_{max} (nm)	32		
Total quality estimate (GNOM)(2)	0.70		
Porod volume estimate, Vp (nm3)	684.2		
**Molecular weight determination**			
Theoretical MW from the sequence + glycans, kDa	120		
From MoW (kDa)(3)	208.5		
From Vc (kDa)(4)	267.8		
Volume of correlation (Vc)	1660		
From Size & Shape (kDa)(5)	309.7		
**Atomic modelling**			
Model	Complete glycosylated dimer of MUC2-C		
CRYSOL			
Max. order of harmonics	90		
χ^2	1.9		
FoXS server			
χ^2	1.5		
**Software**			
SAXS data reduction	BsxCuBE / CHROMIXS (6)		
Data processing	Primusqt - ATSAS package (v.3.0.0) (7, 8)		
Ab initio model	DAMMIN (9)		
Model validation, averaging and final refinement	DAMAVER (10)		
Calculation of theoretical intensity and comparison with SAXS data	CRYSOL (11) / FoXS server (12)		
3D graphics representations	UCSF ChimeraX (v1.2.5) (13)		
SASBDB entry	SASDPL4		

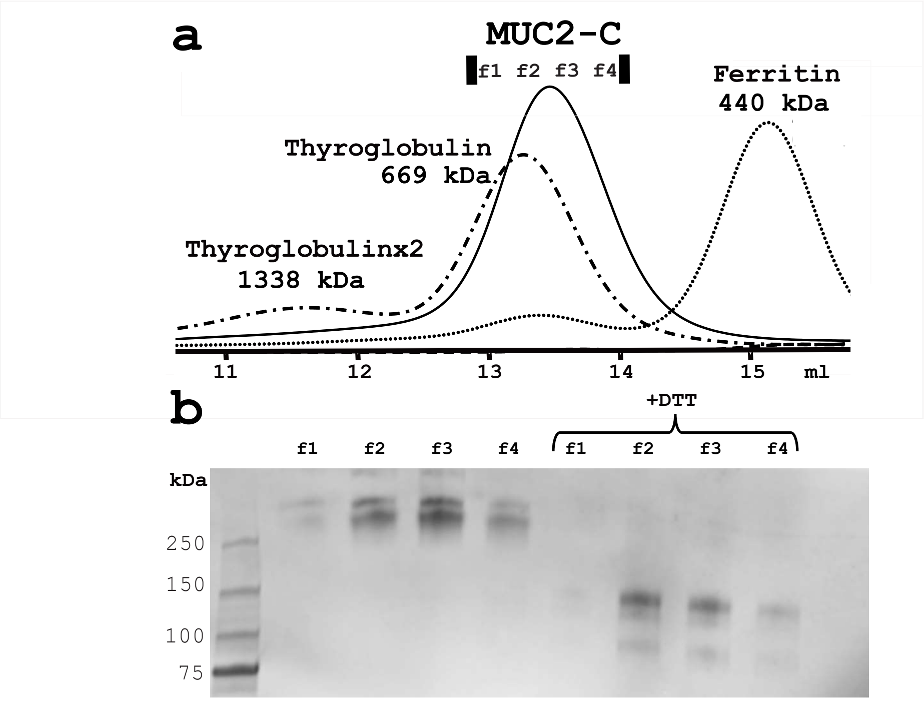

Supplementary Fig. S1. Purification of MUC2 C-terminal recombinant protein. a Size exclusion of the of the MUC2-C recombinant protein using Superose 6 compared with standard thyroglobulin and ferritin. **b** SDS-PAGE, non-reduced and DTT reduced, of the four fractions obtained during purification. The gel filtration and SDS-PAGE was repeated at least three times.

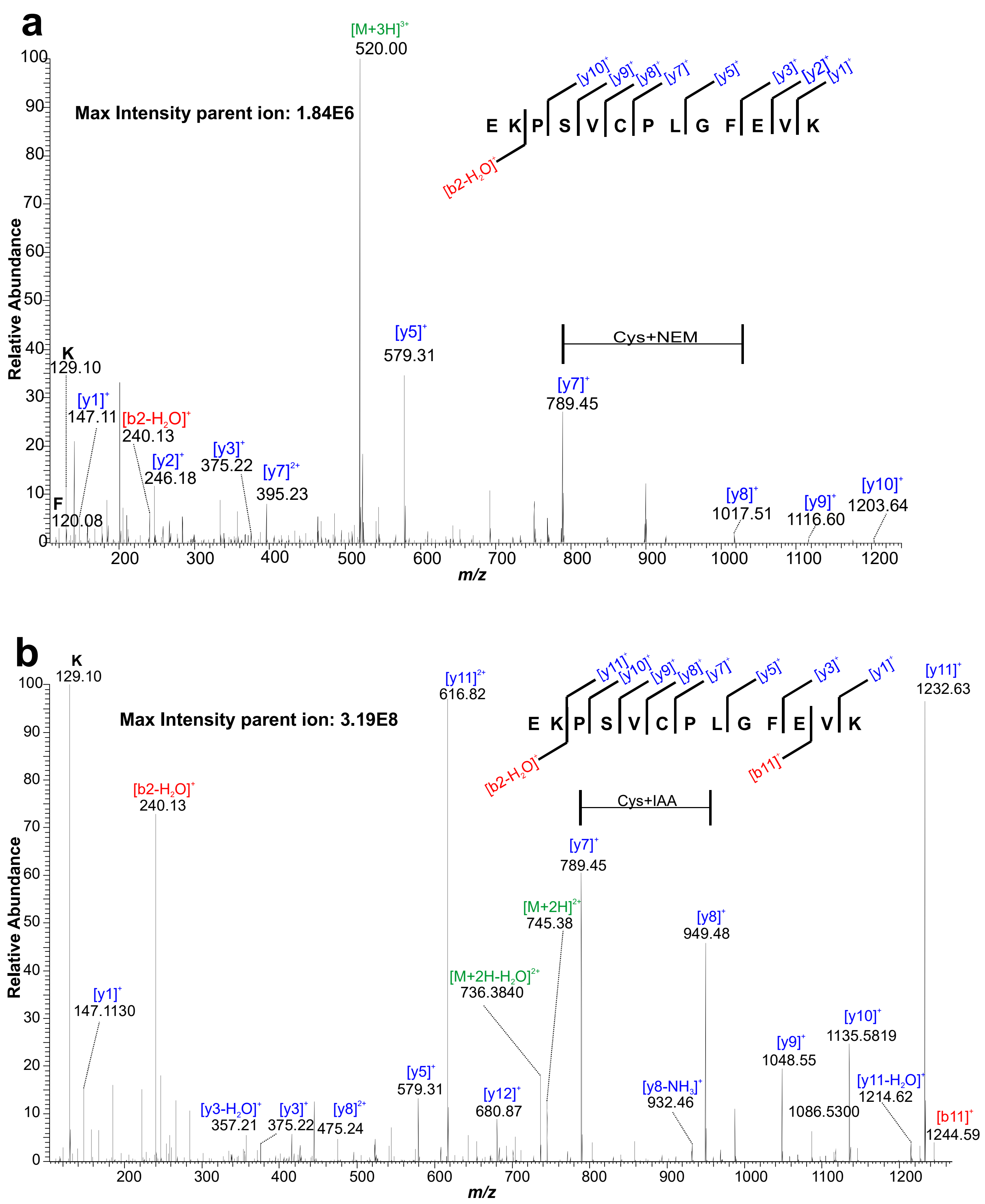

Supplementary Fig. S2. MS2 spectra from one peptide for the differential alkylation approach to detect the presence of free cysteines. The N-ethylmaleimide (**a**) and iodoacetamide (**b**)-labelled spectra of the peptide covering amino acids 4894-4906 in MUC2 are shown. b-ions are labelled in red and y-ions are labelled in blue whereas the parent ions are labelled in green. Immonium ions are labelled with the symbol of the respective amino acid residue. **a** The MS2 spectrum of the parent ion $[M+3H]^{3+}$ 519.94. The N-ethylmaleimide-labeled Cys residue is highlighted by the marked distance between the y7 and y8 ion with Cys-NEM. The maximum intensity of the parent ion was 1.84E6. **b** The MS2 spectrum of the parent ion $[M+2H]^{2+}$ 745.39. The iodoacetamide-label of the Cys residue is highlighted by the marked distance between the y7 and y8 ion with Cys-IAA. The maximum intensity of the parent ion was 3.19E8.

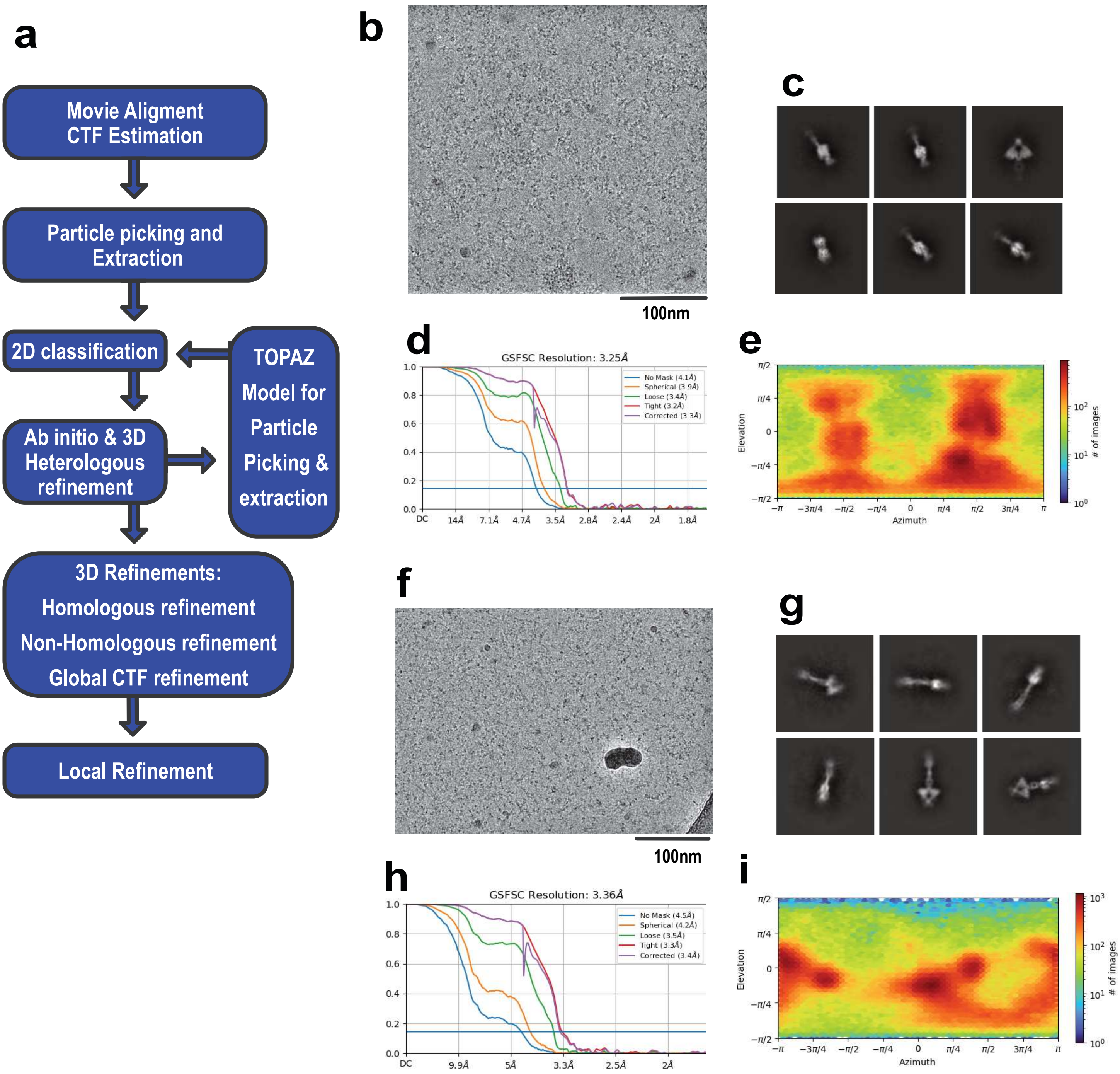

Supplementary Fig. S3. Structure and resolution determination of glycosylated and deglycosylated MUC2-C. **a** Flowchart summary of Cryo-EM processing steps from frame alignment to 3D reconstruction. **b** Example of micrograph collected for MUC2-C deglycosylated. Scale bar indicated. **c** Representative 2D class averages for deglycosylated MUC2-C. **d** Fourier shell correlations (FSC) for the final density map with a resolution of 3.25 Å for deglycosylated MUC2-C. **e** Per-particle distribution over azimuth and elevation angles for the final density map for deglycosylated MUC2-C. **f** Example of micrograph collected for glycosylated MUC2-C. Scale bar indicated. **g** Representative 2D class averages for glycosylated MUC2-C. **h** Fourier shell correlations (FSC) for the final density map with a resolution of 3.36 Å for glycosylated MUC2-C. **i** Per-particle distribution over azimuth and elevation angles for the final density map for glycosylated MUC2-C.

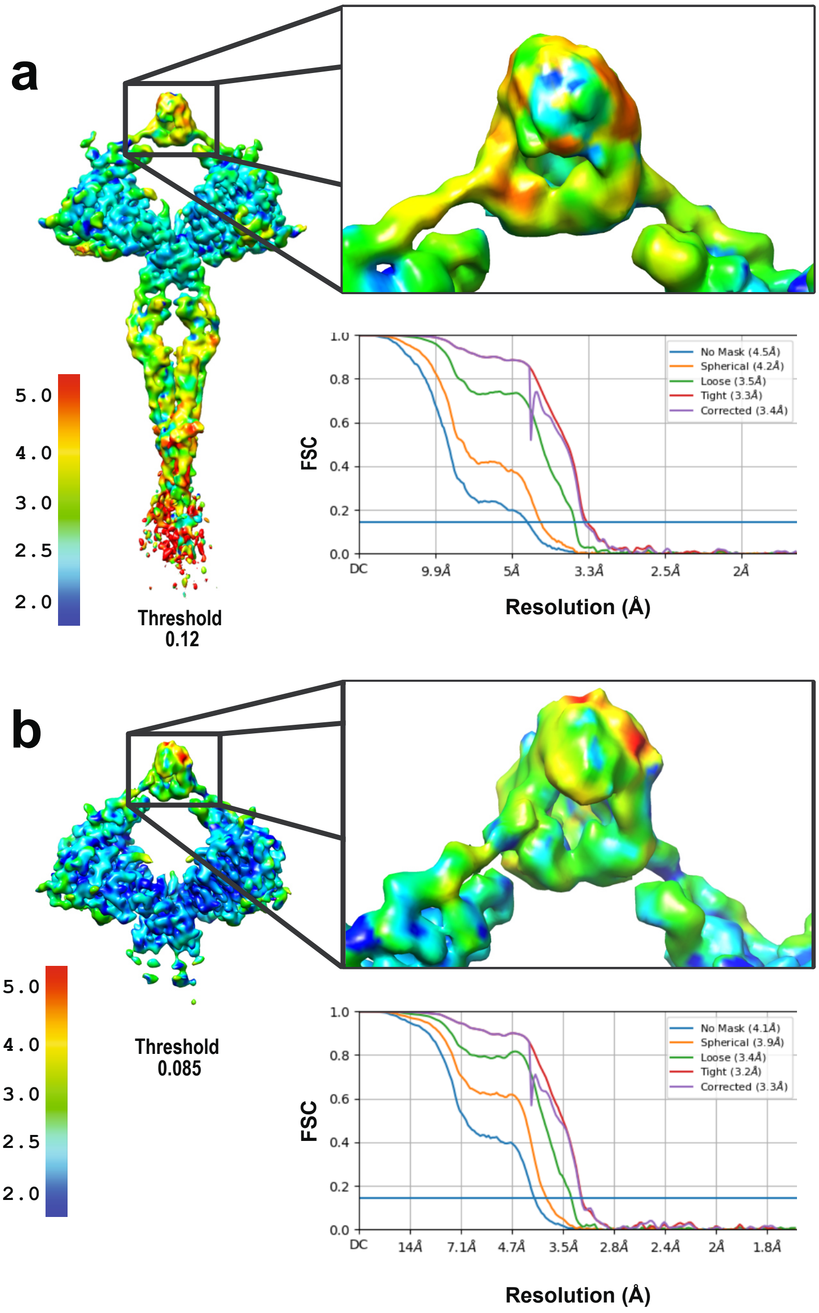

Supplementary Fig. S4. Cryo-EM density maps colored by local resolution. **a** Map of the glycosylated MUC2-C at 3.36 Å. Enlarged N-lobe of the WVCN domain. In the right panel, Fourier Shell Correlations (FSC) for the final density map. **b** Map of the deglycosylated MUC2-C at 3.25 Å. Enlarged N-lobe of the WVCN domain. In the right panel, Fourier Shell Correlations (FSC) for the final density map.

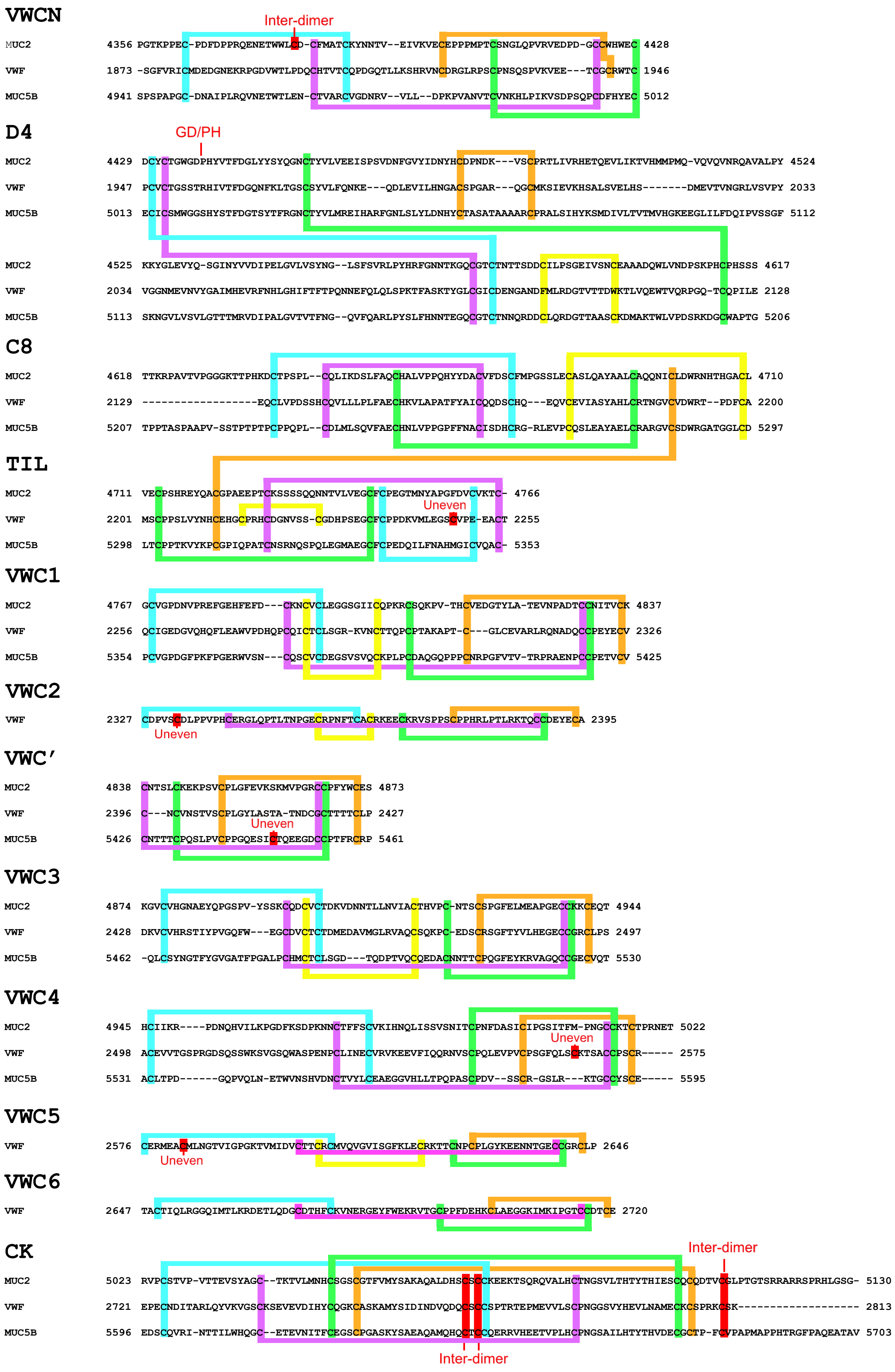

Supplementary Fig. S5. Sequence alignment of the C-terminal parts of the human MUC2, VWF, and MUC5B. The sequences of the large central exon with the PTS sequences of the MUC2 and MUC5B mucins varies between individuals and databases giving different amino acid numbers although the C-terminal sequences are the same. The numbers given are according the that of the Mucin Database, www.medkem.gu.se/mucinbiology/databases, where the MUC2 is from reference (14) (NCBI reference: MH593786.1), human VWF is according to Uniprot P04275 (NCBI reference: NM000552.4), and the MUC5B (UniProtKB/Swiss-Prot: Q9HC84.2, the actual amino acid number vary depending on variabilities in the central PTS sequences).

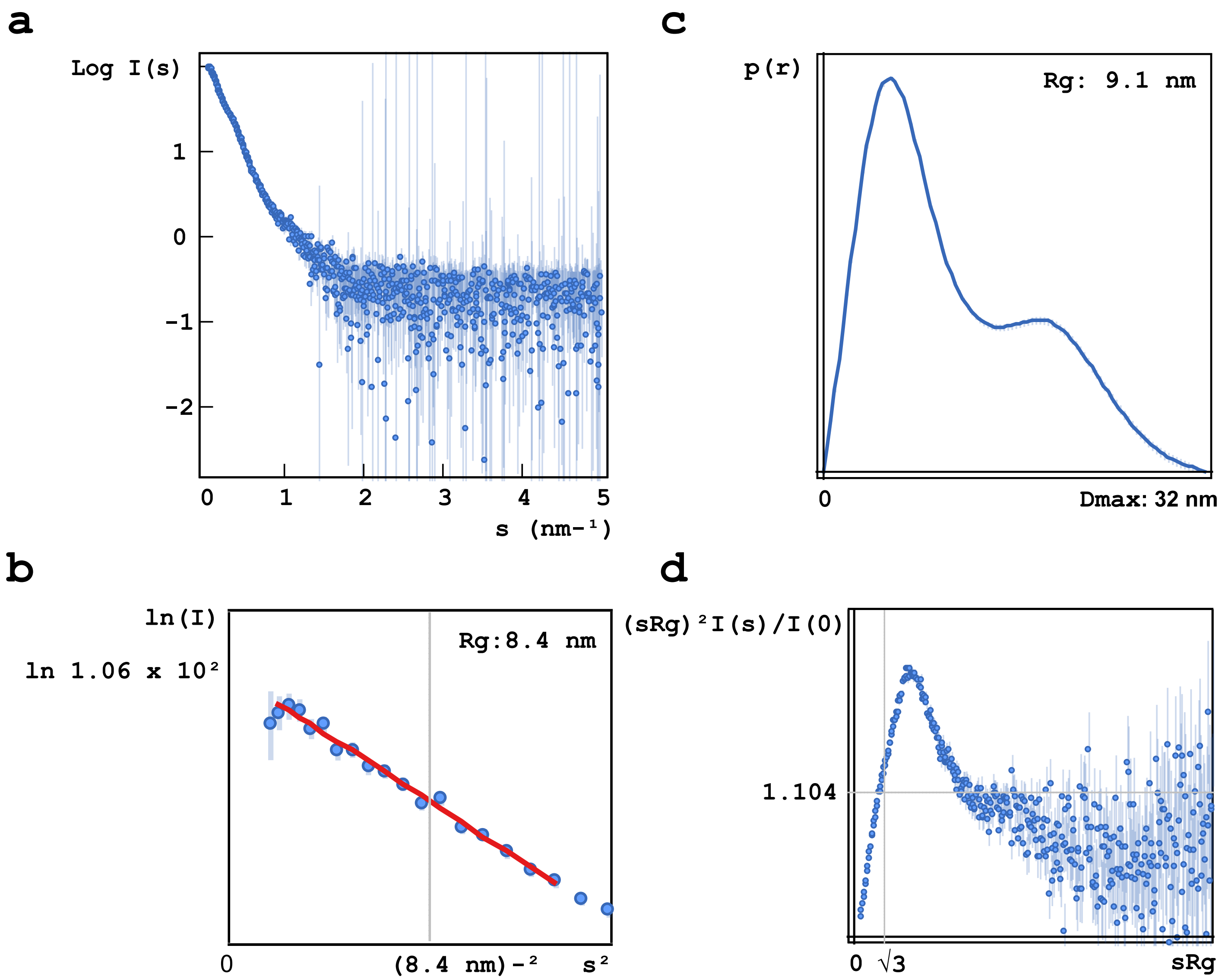

Supplementary Fig. S6. SAXS results of the recombinant MUC2-C. **a** Scattering curve. **b** Guinier plot and radius of gyration (Rg). The calculated scattering curve from the complete glycosylated dimer structure of MUC2-C (Fig. 4B) agrees closely with the SAXS experimental data (SI Fig. S3A) with a $\chi 2$ value of 1.9 using CRYSOL (11) and 1.5 with FoXS server (12). **c** Pr distribution function and Dmax. **d** Kratky plot.

References

1. Petoukhov, M. V., Konarev, P. V., Kikhneya, A. G., & Sverguna, D. I. (2007) *J. Appl. Crystallography* **40**, s223-s228.
2. Svergun, D. I. (1992) *J. Appl. Crystallography* **25**, 495-503.
3. Fischer, H., De Oliveira Neto, M., Napolitano, H. B., Polikarpov, I., & Craievich, A. F. (2010) *J. Appl. Crystallography* **43**, 101-109.
4. Rambo, R. P. & Tainer, J. A. (2013) *Nature* **496**, 477-481.
5. Franke, D. & Svergun, D. (2009) *J. Appl. Crystallography* **42**, 342-346.
6. Panjkovich, A. & Svergun, D. I. (2018) *Bioinformatics* **34**, 1944-1946.
7. Konarev, P. V., Volkov, V. V., Sokolova, A. V., Koch, M. H. J., & Svergun, D. I. (2003) *J. Appl. Crystallography* **36**, 1277-1282.
8. Manalastas-Cantos, K., Konarev, P. V., Hajizadeh, N. R., Kikhney, A. G., Petoukhov, M. V., Molodenskiy, D. S., Panjokovich, A., Haydyn, D., Mertens, T., Gruzinov, A. *et al.* (2021) *J. Appl. Crystallography* **54**, 343-355.
9. Svergun, D. I. (1999) *Biophysical Journal* **76**, 2879-2886.
10. Volkov, V. V. & Svergun, D. I. (2003) *J. Appl. Crystallography* **36**, 860-864.
11. Svergun, D., Barberato, C., & Koch, M. H. J. (1995) *J. Appl. Crystallography* **28**, 768-773.
12. Schneidman-Duhovny, D., Hammel, M., Tainer, J., & Sali, A. (2013) *Biophysical Journal* **105**, 962-974.
13. Pettersen, E. F., Goddard, T. D., Huang, C. C., Meng, E. C., Couch, G. S., Croll, T. I., Morris, J. H., & Ferrin, T. E. (2021) *Protein Science* **30**, 70-82.
14. Svensson, F., Lang, T., Johansson, M. E. V., & Hansson, G. C. (2018) *Scientific Reports* **8**, 17503.
