# Supplementary material for: The intestinal MUC2 mucin C-terminus is stabilized by an extra disulfide bond in comparison to von Willebrand factor and other gel-forming mucins
Source: Nat Commun. 2023 Apr 8;14:1969. doi: 10.1038/s41467-023-37666-8 (PMC10082768; doi:10.1038/s41467-023-37666-8)
Supplement: Supplementary file 2 — Reporting Summary [file 41467_2023_37666_MOESM2_ESM.pdf]

nature portfolio

Corresponding author(s): Gunnar C. Hansson

Last updated by author(s): Jan 2, 2023

Reporting Summary

Nature Portfolio wishes to improve the reproducibility of the work that we publish. This form provides structure for consistency and transparency in reporting. For further information on Nature Portfolio policies, see our Editorial Policies and the Editorial Policy Checklist.

Statistics

For all statistical analyses, confirm that the following items are present in the figure legend, table legend, main text, or Methods section.

n/a	Confirmed	
☒	☐	The exact sample size (n) for each experimental group/condition, given as a discrete number and unit of measurement
☒	☐	A statement on whether measurements were taken from distinct samples or whether the same sample was measured repeatedly
☐	☒	The statistical test(s) used AND whether they are one- or two-sided<br>*Only common tests should be described solely by name; describe more complex techniques in the Methods section.*
☒	☐	A description of all covariates tested
☒	☐	A description of any assumptions or corrections, such as tests of normality and adjustment for multiple comparisons
☒	☐	A full description of the statistical parameters including central tendency (e.g. means) or other basic estimates (e.g. regression coefficient) AND variation (e.g. standard deviation) or associated estimates of uncertainty (e.g. confidence intervals)
☒	☐	For null hypothesis testing, the test statistic (e.g. F, t, r) with confidence intervals, effect sizes, degrees of freedom and P value noted<br>*Give P values as exact values whenever suitable.*
☒	☐	For Bayesian analysis, information on the choice of priors and Markov chain Monte Carlo settings
☒	☐	For hierarchical and complex designs, identification of the appropriate level for tests and full reporting of outcomes
☒	☐	Estimates of effect sizes (e.g. Cohen's d, Pearson's r), indicating how they were calculated

Our web collection on statistics for biologists contains articles on many of the points above.

Software and code

Policy information about availability of computer code

Data collection	CryoEM data was collected in a Titan Krios G2 transmission electron microscope (operated at 300kV) by the National SciLife Facility in Stockholm.<br>Small Angle X-ray Scattering data was collected at the beamline BM29 of the European Synchrotron Radiation Facility (ESRF) in Grenoble, France, by the local contact.<br>The collection parameters for both methods are described in material and methods section and in the supplementary tables S1 and S2 respectively.
Data analysis	Cryo-EM data was analyzed with cryoSPARC (v3.2), the structures were build and refined using Coot (v0.9.6), structures were also refined using Real Space Refinement from Phenix (v1.19.2-4158) and the structures were validated by Molprobity (v4.4) from Phenix.<br>The complete model of MUC2-C was obtained combining the experimental CryoEM data, a model prediction produced by AlphaFold2 at Google Collaboratory (Colab) notebook, the construction of missing loops using Modloop(v main.44475997) and the addition of glycosylations using CHARMM-GUI (v3.7).<br>SAXS data was analyzed using the ATSAS package (v3.0.0) and the FoXS online server.<br>Structural analysis and figures preparation were done using Pymol (v2.4.1) and UCSF Chimera (v1.15) and UCSF ChimeraX (v1.2.5).<br>Mass spectra were analyzed by the Mascot (v.2.3.2), Thermo XCalibur (2.2), and Peaks (v. 8.5).<br>Structural prediction was done by Alphafold (2.1) using an in-house installation.

For manuscripts utilizing custom algorithms or software that are central to the research but not yet described in published literature, software must be made available to editors and reviewers. We strongly encourage code deposition in a community repository (e.g. GitHub). See the Nature Portfolio guidelines for submitting code & software for further information.

Data

Policy information about availability of data

All manuscripts must include a data availability statement. This statement should provide the following information, where applicable:

- Accession codes, unique identifiers, or web links for publicly available datasets
- A description of any restrictions on data availability
- For clinical datasets or third party data, please ensure that the statement adheres to our policy

The protein sequences were MUC2 from Svensson et al. (2018) Scientific Reports 8, 17503(NCBI reference: MH593786.1), VWF (Uniprot P04275, NCBI reference NM_000552.4), and the MUC5B (UniProtKB/Swiss-Prot: Q9HC84.2) The cryo EM structures and maps have been deposited in The Worldwide Protein Data Bank archive [http://www.wwpdb.org/] under accession codes 7QCL and [https://doi.org/10.2210/pdb7QCL/pdb], and 7QCU [https://doi.org/10.2210/pdb7QCU/pdb]. The previously published PDB structures referred in the text are found under accession codes 6RBF [https://doi.org/10.2210/pdb6RBF/pdb] and 6FWN [https://doi.org/10.2210/pdb6FWN/pdb]. The cryo EM maps are deposited to the Electron Microscopy Data Bank (https://www.ebi.ac.uk/emdb/) under accession codes EMD-13896 [https://www.ebi.ac.uk/emdb/EMD-13896] and EMD-13899 [https://www.ebi.ac.uk/emdb/EMD-13899]. SAXS data are deposited to the Small Angle Scattering Biological Data Bank [https://www.sasbdb.org/] under accession code SASDPL4 [https://www.sasbdb.org/data/SASDPL4]. The complete model of MUC2-C including the glycosylations will be available at the SASBDB and at www.medkem.gu.se/mucinbiology/structures. The authors declare that all relevant data supporting the findings of this study are available within the paper, its supplementary information files, and at the stated open depositories.

Human research participants

Policy information about studies involving human research participants and Sex and Gender in Research.

Reporting on sex and gender	n/a
Population characteristics	n/a
Recruitment	n/a
Ethics oversight	n/a

Note that full information on the approval of the study protocol must also be provided in the manuscript.

Field-specific reporting

Please select the one below that is the best fit for your research. If you are not sure, read the appropriate sections before making your selection.

☒ Life sciences ☐ Behavioural & social sciences ☐ Ecological, evolutionary & environmental sciences

For a reference copy of the document with all sections, see nature.com/documents/nr-reporting-summary-flat.pdf

Life sciences study design

All studies must disclose on these points even when the disclosure is negative.

Sample size	For CryoEM data the number of micrographs and particles stated in Supplementary data. For SAXS data the number of frames collected and average is also stated in the Supplementary data.
Data exclusions	The total particles and the used particles are stated in Table S1. The total frames and the used ones from the SEC-SAXS data are stated in Table S2.
Replication	Data were collected from several grids and several sessions, all showing the same particles. All data for deeper analysis was collected during one microscopy session or synchrotron session. This is how single-particle Cryo-EM microscopy and Small Angle X-ray Scattering is performed.
Randomization	Not applicable to structural work. The reconstruction of 3D electron density maps and SAXS data analysis were performed using established methods and particles classified and collected using computer software over many grids.
Blinding	Not applicable to structural work. The reconstruction of 3D electron density maps and SAXS data analysis were performed using established methods using computer software and not persons interpreting individual particles.

Reporting for specific materials, systems and methods

We require information from authors about some types of materials, experimental systems and methods used in many studies. Here, indicate whether each material, system or method listed is relevant to your study. If you are not sure if a list item applies to your research, read the appropriate section before selecting a response.

Materials & experimental systems

n/a	Involved in the study
☒	☐ Antibodies
☐	☒ Eukaryotic cell lines
☒	☐ Palaeontology and archaeology
☒	☐ Animals and other organisms
☒	☐ Clinical data
☒	☐ Dual use research of concern

Methods

n/a	Involved in the study
☒	☐ ChIP-seq
☒	☐ Flow cytometry
☒	☐ MRI-based neuroimaging

Eukaryotic cell lines

Policy information about cell lines and Sex and Gender in Research

Cell line source(s)	The protein sample used in this project was produced in CHO-S cells (R80007, ThermoFisher Scientific). CHO-S are adapted chinese hamster ovary (CHO K1) to serum-free suspension culture.
Authentication	These mammalian cells are controlled by morphology and growth characteristics. The expression were carried out at the Mammalian Protein Expression Core facility at Goteborg University. They work with commercial cell lines.
Mycoplasma contamination	The Mammalian Protein Expression Core facility express protein in commercial cell lines and they perform routinely checks of possible mycoplasma contamination four times a year.
Commonly misidentified lines (See ICLAC register)	No commonly misidentified cell line was used.
